# Influences of quality of maternal care and environmental enrichment on associative memory function in rats with early life lead exposure

**DOI:** 10.1002/brb3.70040

**Published:** 2024-09-18

**Authors:** Jay S. Schneider, Courtney Williams, Shamaila Zafar, Jaehyun Joo, Blanca. E. Himes

**Affiliations:** ^1^ Department of Pathology and Genomic Medicine Thomas Jefferson University Philadelphia Pennsylvania USA; ^2^ Center of Excellence in Environmental Toxicology, Perelman School of Medicine University of Pennsylvania Philadelphia Pennsylvania USA; ^3^ Department of Biostatistics, Epidemiology and Informatics, Perelman School of Medicine University of Pennsylvania Philadelphia Pennsylvania USA

**Keywords:** environmental enrichment, lead exposure, maternal care, memory

## Abstract

**Introduction:**

Children in low socioeconomic status (SES) communities are at higher risk of exposure to lead (Pb) and potentially more severe adverse outcomes from Pb exposures. While the factors encompassing SES are complex, low SES households often have less enriching home environments and parent–child interactions. This study investigated the extent to which environmental/behavioral factors (quality of maternal care and richness of the postnatal environment) may modify adverse effects from Pb exposure.

**Methods:**

Long‐Evans female rats were randomly assigned to Control (no Pb), Early Postnatal (EPN: birth through weaning), or Perinatal (PERI: 14 days pre‐mating through weaning) Pb exposure groups. From postnatal days (PNDs) 2–9, maternal care behaviors were observed, and dams were classified as low or high maternal care based on amounts of licking/grooming and arched back nursing. At weaning, pups were randomly assigned to enriched or non‐enriched environments. At PND 55, animals began trace fear conditioning and associative memory was tested on days 1, 2, and 10 postconditioning.

**Results:**

Control offspring showed no significant effects of maternal care or enrichment on task performance. Females with EPN‐Pb exposure and males with PERI‐Pb exposure living in the non‐enriched environment and having an LMC mother had significant memory impairments at days 2 and 10 that were not observed in comparably housed animals with HMC mothers. Enriched animals had no deficits, regardless of maternal care status.

**Conclusion:**

These results show the potential for modulatory influences of maternal care and housing environment on protecting against or reversing at least one aspect of Pb‐induced cognitive/behavioral dysfunction.

## Graphical abstract

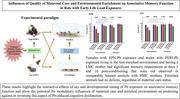

## 1 INTRODUCTION

There are various perinatal environmental and behavioral factors that can influence the development of the brain and subsequent cognitive and behavioral functioning. Among the factors contributing to optimal neurodevelopment are the richness and complexity of the postnatal environment and the quality of caregiving (including the level of cognitive stimulation provided in the home environment), both of which may have a modulatory influence on brain, behavior, and social development (e.g., Bedrosian et al., 2018; Chajes et al., 2022; Giuliani et al., 2019; Hackman et al., 2021; Kuzumaki et al., 2011; Morse et al., 2015). Exposures to environmental neurotoxicants also have modulatory influences on brain, behavior, and social development, often with adverse effects on neural and cognitive/behavioral development (Lidsky & Schneider, 2003).

Developmental exposure to the neurotoxicant lead (Pb) remains an important public health problem due to the persistence of multiple potential routes of exposure (e.g., water, deteriorating paint, topsoil) (Dignam et al., 2019; Wilson et al., 2022), resulting in elevated childhood blood Pb levels (BLLs) and persistent, and potentially permanent, cognitive and behavioral deficits in children (Reuben et al., 2017; Schneider, 2023). Children with elevated BLLs are more likely to be classified as learning disabled (Miranda et al., 2011), perform poorly in school, and underperform on tests of academic achievement (Blackowicz et al., 2016; Evens et al., 2015; Miranda et al., 2007). While all children are potentially at risk from environmental exposure to Pb (Bellinger et al., 1992; Cummins & Goldman, 1992), members of low socioeconomic status (SES) communities are at higher risk of exposure, sustain higher BLLs (White et al., 2016), and have potentially more severe adverse outcomes from these exposures compared to higher SES cohorts (Bellinger, 2008; Harvey et al., 1984; Lansdown et al., 1986; Rutter, 1983; Winneke & Kraemer, 1984). Children from low SES families, in addition to having a higher risk for Pb exposure, are also prone to living in less stimulating environments and are more likely to have less interactive caregivers (Brody et al., 2017; Luby et al., 2013). While the effects of low SES on cognitive and behavioral development are multifactorial, the quality of parental care and interactions (Conger et al., 1994) and the quality of the home environment independently and jointly play important roles (McCartney et al., 2007).

It is important to understand the interactions of environmental and behavioral factors that can potentially exacerbate or mitigate the adverse effects of developmental Pb exposure on cognitive functioning. The impact of one of these important behavioral factors, the quality of maternal care, has not been previously examined as an effect modifier in experimental or clinical studies of Pb neurotoxicity, nor have the combined influences of the quality of maternal care and the quality of the social/housing environment been studied. The quality of early life experiences is potentially very important for influencing outcomes following early neurotoxicant exposures (Cory‐Slechta et al., 2017) and currently, nothing is known about how factors of developmental Pb exposure, quality of maternal care, and quality of the housing environment interact to influence later behavioral/cognitive outcomes. The behavioral studies described in this paper were performed as a first step to understand the extent to which the quality of maternal care and the richness of the postnatal environment, individually or together, may influence the effects of early life Pb exposure (primarily maternally transmitted) on offspring's associative memory function, a cognitive outcome known to be adversely affected by developmental Pb exposure (Anderson et al., 2016). This research set out to address the following questions: In conjunction with early life Pb exposure, can high‐quality maternal care overcome negative effects on cognitive functioning related to living in a non‐enriched environment and is its influence different for males and females? Conversely, can living in an enriched environment overcome detrimental effects on cognitive functioning related to receiving low‐quality maternal care and is its influence different for males and females? In an attempt to answer these basic behavioral questions, we assessed associative memory functioning in male and female offspring of low or high maternal care dams, following either perinatal or early postnatal Pb exposures, and subsequently living in enriched or non‐enriched housing environments. We chose to examine associative memory functioning in this study as our prior research showed this cognitive function to be disrupted in Pb‐exposed rats based on their sex and the timing of Pb exposure, with perinatally Pb‐exposed males and early postnatally Pb‐exposed females having the greatest memory deficits (Anderson et al., 2016).

## 2 MATERIALS AND METHODS

### 2.1 Animals and Experimental Groups

The use of animals was in compliance with NIH Guidelines for the Care and Use of Laboratory Animals and the study was approved by the Institutional Animal Care and Use Committee at Thomas Jefferson University. Long‐Evans hooded male and female rats (Envigo) were purchased at approximately 55–65 days of age and were undisturbed for 7 days after arrival in our facility. Rats were housed in a 12 h:12 h light:dark cycle from 4 a.m. to 4 p.m. and females were randomly assigned to one of three experimental groups: (1) Control (no Pb exposure); (2) Early Postnatal (EPN) Pb exposure, or (3) Perinatal (PERI) Pb exposure. Control females and all males were fed RMH 1000 chow (ICN Biomedicals) ad libitum and Pb‐exposed animals were fed the same chow containing 150 ppm lead acetate (Dyets Inc.). This level of Pb exposure was previously shown by us to result in an environmentally relevant BLL (Verma & Schneider, 2017). For PERI Pb exposure, dams consumed Pb‐containing chow for 14 days prior to mating and continued to consume this chow through weaning. For EPN Pb exposure, dams consumed Pb‐containing chow beginning at parturition and continued to consume this chow through weaning. At postnatal day (PND) 1, all pups in a litter were counted, sexed, culled (i.e., to 5 males and 5 females when possible), and when necessary, were cross‐fostered with dams from the same exposure group in order to maintain litters consisting of 5 males and 5 females for each dam. Once a litter was born, the cage was not changed until PND 10 to prevent any extraneous variables that might influence the dam's engagement in maternal care behaviors.

### 2.2 Maternal Care Behavioral Observations and Scoring

The maternal care behavior observation protocol used was adopted from procedures described in previous studies (e.g., Bredy et al., 2003; Francis et al., 1999; Liu et al., 1997; Myers et al., 1989). Beginning on PND 2 and concluding on PND 9 (i.e., 8 days of observations), dams were observed 3 times per day for 1 h per session. Maternal care behaviors were recorded by the frequency at which they were observed during each session. Assessments were performed at 9 a.m., 1 p.m., and 5 p.m., with the 5 p.m. observation falling during the dark cycle. A red‐light headlamp (0.0–0.2 Lux) was worn by the observer during the dark cycle assessment to allow for the observation of behaviors without disturbing the rats. The observer always entered the room approximately 10 min prior to the start of the observation, quietly shut the door behind them, and waited for the rats to acclimate to their presence. Cages were viewed from all sides to accurately observe maternal care behaviors. Data on maternal care behaviors were collected using a momentary time‐sampling method. Maternal care behaviors were observed and recorded every 3 min during a 1‐h session for a total of 20 observations per dam per session. Based on the rat maternal care/epigenetic programming literature (e.g., Champagne et al., 2003; Kaffman & Meaney, 2007), the maternal care behaviors that were the focus of this study were licking/grooming and arched back nursing. Means and standard deviations for these maternal care behaviors in each exposure group were calculated (Bredy et al., 2003; Francis et al., 1999; Liu et al., 1997). Dams scoring higher than one standard deviation above the mean on arched back nursing and arch back nursing plus licking/grooming were considered to be high maternal care (HMC) dams, whereas dams scoring lower than one standard deviation below the mean on both measures were considered to be low maternal care (LMC) dams. At the conclusion of maternal care ratings (PND 10), all mothers and pups were placed in standard caging and continued to receive Pb‐containing or control diet according to their treatment group until the time of weaning.

### 2.3 Postweaning Housing Conditions

Pups from different dams/cages across different exposure groups were randomly cohoused after weaning to preclude potential cage effects. On PND 21 (weaning), males and females from LMC and HMC dams were randomly assigned to an enriched or non‐enriched housing environment. The enriched environment consisted of a large plastic enclosure (61 cm × 43.5 cm) housing six animals of the same sex and containing a variety of toys, running wheels, climbing and nesting materials, and tunnels that were changed three times per week to provide novelty. The non‐enriched environment consisted of a standard plastic housing enclosure (47.6 cm × 25.9 cm) containing three animals of the same sex with no toys or other external stimuli added to the environment. All postweaning housing enclosures contained approximately a 50:50 mix of Alpha‐Dri:Beta‐Cob bedding material (Shepherd Specialty Papers) to a standard depth of approximately 1 cm.

### 2.4 Trace Fear Conditioning

The trace fear conditioning paradigm is sensitive to detecting associative memory deficits in Pb‐exposed rats based on sex and timing of Pb exposure, with PERI‐Pb‐exposed males and EPN Pb‐exposed females most sensitive to expressing memory deficits, as shown in a previous study (Anderson et al., 2016). Beginning at approximately PND 55, animals were habituated to the fear conditioning chamber, which consisted of a dimly lit sound‐attenuating enclosure containing a test box with a grid floor through which shocks could be delivered, and equipped to provide white background noise (Ugo Basile). Habituation was for 10 min one day prior to the start of fear conditioning. During conditioning trials, animals acclimated to the test box for 2 min and then were presented with a series of six tone‐shock pairs (tone: 3000 Hz, 80 dB for 15 s; shock: 0.8 mA for 1.0 s). Freezing behavior (the absence of all but respiratory movements) was measured every second during the 20 s trace period (time between tone presentation and shock) using Anymaze software (Stoelting, Inc.). Each tone‐shock pairing was followed by a random inter‐trial interval (ITI) that varied between 1 and 3 min. After the last of the six tone‐shock pairings, animals were returned to their home cages. Retention testing then took place 1, 2, and 10 days postconditioning with animals placed back into the same chamber in which they were trained. On each testing day, animals were again acclimated to the chamber for 2 min followed by the presentation of 3 tones for 15 s each, in the absence of foot shock, with pseudorandom ITIs between tone presentations. Freezing was measured by the Anymaze software (with treatment groups randomly coded) every second during the 20 s period following the tone presentation.

For analysis of trace fear conditioning data, and based on our previous data showing sex‐specific effects of Pb exposure in this paradigm (Anderson et al., 2016), female and male animals were considered as different animal models and hence, statistical analyses were performed separately for males and females per endpoint. Missing values were imputed using predictive mean matching (PMM) within the multivariate imputation by chained equations (mice) R package (Stef van Buuren, 2011). To account for imputation uncertainty, we performed multiple imputations that generate multiple imputed data sets (n = 100). We fitted a mixed‐effect model to each of those imputed datasets separately in which a rat‐specific intercept was specified as a random effect. The fitted results were pooled across the models for statistical inference (Toutenburg & Rubin, 1990). Contrast tests were then performed using the estimated marginal means (emmeans) R package (Lenth, 2023) to compare biological conditions of interest. To account for multiple tests, *p* values were adjusted based on the multivariate *t*‐distribution incorporating the same covariance structure as the estimates (Hothorn et al., 2008).

### 2.5 BLL Analyses

At the time of weaning, a random sample of dams from each group was euthanized and blood was collected to evaluate BLLs. When available, random extra pups from the various groups, which were not to be used in any subsequent studies, were also euthanized at weaning and blood was collected for analysis. Blood was collected in an ethylenediaminetetraacetic acid (EDTA)‐containing tube (Sardstedt) and mixed to prevent coagulation. Blood samples were analyzed using a commercial ESA LeadCare II Blood Lead Analyzer (Magellan Diagnostics) based on electrochemical anodic stripping voltammetry (ASV). A total of 50 µL of whole blood was mixed with 250 µL of hydrochloric acid solution (0.34 M) and the mixture was applied to a sensor strip inserted into the LeadCare II Blood Lead Analyzer. After 3 min, the BLLs were reported from the instrument in µg/dL, with a lower sensitivity detection level cutoff value of 3.3 µg/dL (SEM ± 1.5 µg/dL). Differences in BLLs were analyzed within sex across the experimental groups by ANOVA followed by post hoc analysis using Tukey's multiple comparison test.

## 3 RESULTS

### 3.1 Maternal Care Characteristics

A total of 219 dams were assessed for maternal care characteristics: 49 were non‐Pb‐exposed Controls, 90 were in the EPN Pb group, and 80 were in the PERI Pb group. The numbers of animals in each treatment group and designated as LMC, HMC, or Other are shown in Table 1. In the Control group, 26.5% of dams were designated LMC, compared to 15.5% in the EPN group and 16.3% in the PERI group. The Control group had 20.4% HMC dams, compared to 16.7% in the EPN Pb group and 13.8% in the PERI Pb group. While the percent of dams characterized as LMC, HMC, and Other were slightly different in the Control group compared to the Pb‐exposed groups, perhaps due to the overall smaller size of the Control group, the numbers of LMC and HMC dams in the different groups were not statistically significantly different from each other (Fisher's exact test, *p* = .33) (Table 1).

**TABLE 1:** Numbers (percent) of dams categorized as low maternal care (LMC), high maternal care (HMC), or other (not meeting criteria for LMC or HMC).

Groups	Total # dams	Number (percent) LMC	Number (percent) HMC	Number (percent) other
Control	49	13 (26.5)	10 (20.4)	26 (53.1)
EPN Pb	90	14 (15.5)	15 (16.7)	61 (67.8)
PERI Pb	80	13 (16.3)	11 13.8)	56 (70.0)

*Note*: No statistically significant group differences were observed.

Abbreviations: Control, no Pb exposure; EPN, early postnatal Pb exposure; PERI, perinatal Pb exposure.

### 3.2 BLLs

Lead exposure of dams in the PERI Pb exposure group prior to mating and during gestation did not result in any preterm births or in smaller litter sizes compared to Control animals without Pb exposure and those in the EPN Pb exposure group. Likewise, the natural variability in the numbers of male and female pups in any particular litter was not different across the different treatment groups, nor was the frequency of cross‐fostering.

BLLs from dams and from pups (collected at the time of weaning) are shown in Table 2. As expected, all Control animals had BLLs below the level of detection of the LeadCare II system. All Pb‐exposed animals, including pups, had elevated BLLs compared to Controls. There were no statistically significant differences in BLLs related to maternal care characteristics of dams or timing of Pb exposure. There were no statistically significant differences in BLLs in Pb‐exposed pups, regardless of sex, developmental timing of Pb exposure, or maternal care characteristics of mothers (Table 2).

**TABLE 2:** Blood lead (Pb) levels for all study groups.

	Dams		Pups (females)		Pups (males)	
Group	*N*	Blood lead levels (µg/dL)	*N*	Blood lead levels (µg/dL)	*N*	Blood lead levels (µg/dL)
**PERI LMC**	13	7.1 ± 0.6	13	7.1 ± 0.6	6	7.5 ± 0.8
**PERI HMC**	17	8.2 ± 0.4	17	8.2 ± 0.4	11	8.2 ± 0.7
**PERI Other**	8	7.0 ± 0.7	8	7.0 ± 0.7	11	6.0 ± 0.4
**EPN LMC**	13	5.7 ± 0.6	7	8.7 ± 1.1	10	8.6 ± 0.9
**EPN HMC**	11	5.9 ± 0.6	9	7.0 ± 0.8	10	6.6 ± 1.1
**EPN Other**	17	6.6 ± 0.5	11	7.8 ± 0.7	11	5.8 ± 0.6

*Note*: Blood lead (Pb) levels are expressed as mean ± SEM. All Control animals had non‐detectable levels of Pb in the blood (<3.3 µg/dL, the detection limit of the LeadCare II Analyzer). No statistically significant group differences were observed.

Abbreviations: EPN, early postnatal Pb exposure; HMC, high maternal care; LMC, low maternal care; Other, did not meet criteria for LMC or HMC; PERI, perinatal Pb exposure.

### 3.3 Trace Fear Conditioning

**FIGURE 1:**
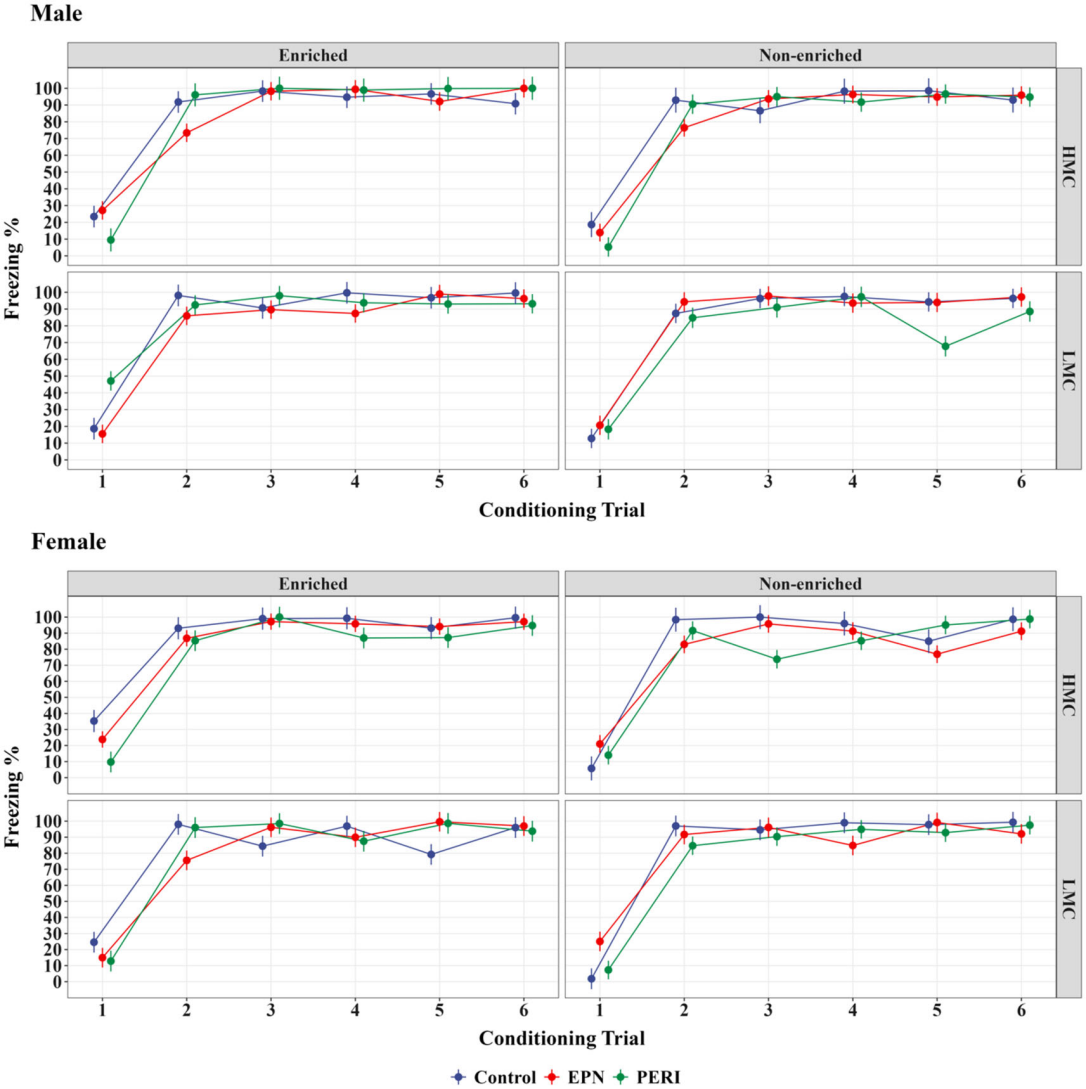
During the conditioning phase of the trace fear conditioning test, all groups of animals quickly learned the tone‐shock association and performed similarly over the six tone‐shock pairing trials, as demonstrated by the lack of significant differences in freezing responses across the various test groups. EPN, early postnatal Pb exposure; HMC, high maternal care; LMC, low maternal care; PERI, perinatal Pb exposure.

Learning curves were examined to assess whether there were any effects of Pb exposure, sex, quality of maternal care, and/or enrichment housing on freezing responses over the course of the six conditioning trials in the trace fear conditioning paradigm. No statistically significant differences were observed between any of the experimental groups in their freezing responses during the conditioning trials on this test (Figure 1; Table S1).

**FIGURE 2:**
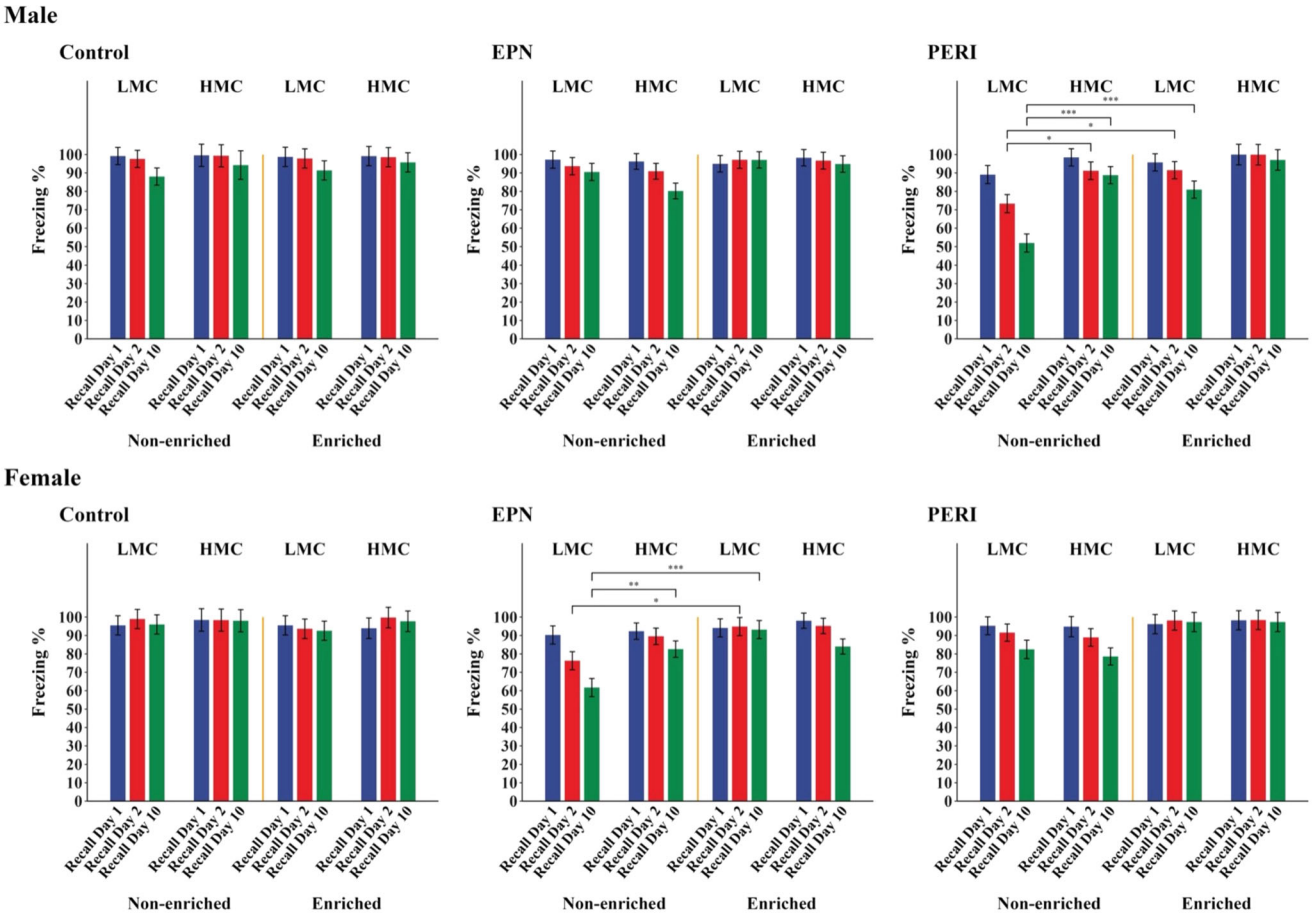
Effects of quality of maternal care and environmental enrichment on associative memory function. A decrease in percent freezing during the trace period from day 1 to day 10 postconditioning is indicative of an associative memory impairment. In males, only PERI LMC non‐enriched animals (*N* = 10) had a significant impairment compared to similar Control non‐Pb‐exposed animals (*N* = 10). Within the PERI LMC group, there were significant differences in memory performance at day 2 and day 10 postconditioning between enriched (*N* = 8) and non‐enriched animals (*N* = 10). In non‐enriched PERI males, there were also significant differences in memory performance at day 2 and day 10 postconditioning between LMC (*N* = 10) and HMC (*N* = 6) groups. In females, only EPN LMC non‐enriched animals (*N* = 9) had a significant impairment compared to similar Control non‐Pb‐exposed animals (*N* = 8). Within the EPN LMC group, there were significant differences in memory performance at day 2 and day 10 postconditioning between enriched (*N* = 9) and non‐enriched (*N* = 9) animals. In non‐enriched EPN females, there was also a significant difference in memory performance at day 10 postconditioning between LMC (*N* = 9) and HMC (*N* = 11) groups. *p < .05; **p < .01; ***p < .001.

In contrast to the lack of differences observed during conditioning trials, significant group differences in freezing responses were observed during memory testing. In PERI‐Pb‐exposed animals, only PERI Pb‐exposed males from an LMC mother and that subsequently lived in a non‐enriched environment (*N* = 9) had statistically significantly different memory performance compared to similar Controls (*N* = 10) (Table 3). These animals began to show a significant impairment in associative memory, as measured by a statistically significant decrease in freezing response, at day 2 of postconditioning testing (*p* = .001 vs. Control) that persisted at day 10 testing (*p* < .001) (Figure 2, Table 3, Figure S1). In comparison, PERI Pb‐exposed males from an HMC mother that also lived in the non‐enriched environment (*N* = 10) showed no memory deficits (i.e., no change in freezing responses) at any of the postconditioning test days (Figure 2, Figure S1). PERI Pb‐exposed males from a LMC mother and that lived in an enriched environment (*N* = 10) had a slight decrease in percent of freezing at day 10 postconditioning. However, their freezing response, while not significantly different from that obtained from Controls (*N* = 8), was significantly different from the LMC, non‐enriched animals (Figure 2, Figure S1). Males with EPN Pb‐exposure showed no changes in their freezing responses across any of the postconditioning test days and thus no evidence of an associative memory deficit regardless of whether they had an LMC or HMC mother or whether they lived in enriched or non‐enriched environments.

**TABLE 3:** Associative memory function (percent freezing) comparison between PERI Pb‐exposed and Control LMC males.

		Control	PERI	PERI vs. Control			
Environmental enrichment	Recall day	Mean (SE)	Mean (SE)	Difference^a^ (SE)	*t* statistic	df	Adjusted *p*‐value
Enriched	1	98.75 (5.22)	95.75 (4.67)	−3.00 (7.01)	−0.43	486	0.96
	2	97.87 (5.22)	91.50 (4.67)	−6.37 (7.01)	−0.91	486	0.72
	10	91.37 (5.22)	80.95 (4.67)	−10.42 (7.01)	−1.49	486	0.34
Non‐enriched	1	99.20 (4.67)	89.11 (4.93)	−10.09 (6.79)	−1.49	486	0.34
	2	97.65 (4.67)	73.33 (4.93)	−24.32 (6.79)	−3.58	486	1.1e‐03
	10	88.05 (4.67)	51.94 (4.93)	−36.11 (6.79)	−5.32	486	5.1e‐07

*Note*: Numbers represent freezing response (i.e., mean (SE) percent of time freezing). Control enriched: *N* = 10; Control non‐enriched: *N* = 9; PERI enriched: *N* = 10; PERI non‐enriched: *N* = 9.

Abbreviations: df, degrees of freedom; SE, standard error.

^a^ A vs. B = A − B.

In females, although there were some trends toward decreased freezing responses at day 10 in PERI Pb‐exposed animals, a statistically significant deficit in associative memory was only observed in EPN Pb‐exposed females from LMC mothers and that lived in the non‐enriched environment (*N* = 9) (Figure 2; Table 4; Figure S1). These animals had a significant memory impairment at day 2 of postconditioning testing (*p* = .005 vs. Control (*N* = 8)) that persisted at day 10 testing (*p* < .001). Females from HMC mothers and that lived in the non‐enriched environment (*N* = 11) showed no significant memory deficits compared to Controls (*N* = 6). Likewise, EPN Pb‐exposed females that lived in the enriched environment showed no memory deficits regardless of whether their mothers were characterized as LMC (*N* = 9) or HMC (*N* = 13) (Figure 2; Table S2). Females with PERI Pb‐exposure showed no evidence of an associative memory deficit regardless of whether they had an LMC or HMC mother and whether they lived in an enriched or non‐enriched environment (Figure 2; Table S2; Figure S1).

**TABLE 4:** Memory function comparison between EPN Pb‐exposed and Control LMC females.

		Control	EPN	EPN vs. Control			
Environmental enrichment	Recall day	Mean (SE)	Mean (SE)	Difference^a^ (SE)	*t* statistic	df	Adjusted *p*‐value
Enriched	1	95.50 (5.22)	94.11 (4.93)	−1.39 (7.18)	−0.19	486	1.00
	2	93.62 (5.31)	94.83 (4.93)	1.21 (7.24)	0.17	486	1.00
	10	92.56 (5.22)	93.17 (4.93)	0.60 (7.18)	0.08	486	1.00
Non‐enriched	1	95.50 (5.22)	90.28 (4.93)	−5.22 (7.18)	−0.73	486	0.83
	2	98.94 (5.22)	76.28 (4.93)	−22.66 (7.18)	−3.16	486	5.0e‐03
	10	96.00 (5.22)	82.44 (4.93)	−34.28 (7.18)	−4.77	486	7.2e‐06

*Note*: Numbers represent freezing response (i.e., mean (SE) percent of time freezing). Control enriched: *N* = 8; Control non‐enriched: *N* = 8; EPN enriched: *N* = 9; EPN, non‐enriched: *N* = 9.

Abbreviations: df, degrees of freedom; SE, standard error.

^a^ A vs. B = A − B.

## 4 DISCUSSION

Early life exposure to the neurotoxicant Pb remains a critical public health problem in the United States and around the world (Larsen & Sanchez‐Triana, 2023), with Pb being 1 of the 10 chemicals listed by the World Health Organization as being of major public health concern (WHO 2023). In the United States, Pb neurotoxicity is experienced more frequently by children in low SES households, where there is a higher risk for Pb exposure, a tendency for less stimulating environments, having less consistent or enriching parent–child interactions (Brody et al., 2017; Luby et al., 2013), and having greater magnitude Pb‐induced neurodevelopmental deficits than children living in higher SES households with similar exposures (Bellinger, 2008; Harvey et al., 1984; Lansdown et al., 1986; Rutter, 1983; Winneke & Kraemer, 1984). To the best of our knowledge, the extent to which the complex social factors of quality of maternal care and the richness of the postnatal environment may interact to modulate the brain's response to Pb exposure and modify cognitive outcomes have not previously been studied. Thus, this study examined for the first time, the impact of the quality of maternal care together with the complexity of the postweaning environment on associative memory function, a cognitive outcome previously shown by us to be disrupted in a sex‐dependent manner by different early life Pb exposures (Anderson et al., 2016; Verma & Schneider, 2017). We observed that in non‐Pb‐exposed rats, males or females, neither the quality of maternal care nor housing in an enriched or non‐enriched environment had statistically significant modulatory effects, either positive or negative, on associative memory function (i.e., percent freezing) in the trace fear conditioning paradigm that was used. Environmental enrichment has been previously suggested to have positive effects on some tests of learning and memory, and on hippocampal neurogenesis, synaptic transmission, and various transcriptional changes in various brain regions (Bekinschtein et al., 2011; Mora‐Gallegos & Fornaguera, 2019; Pena et al., 2009; Rampon et al., 2000). However, the parameters of the trace fear conditioning paradigm that we used in this study were designed to primarily detect memory deficits in Pb‐exposed animals and thus due to ceiling effects, normal animals raised in the enriched environment and tested using the same parameters would be unlikely to show an improvement from normal responding. Animals reared in social isolation or impoverished environments have also been reported to have decreased performance in some learning and memory tasks (Bekinschtein et al., 2011; Mora‐Gallegos & Fornaguera, 2019; Pena et al., 2009). The non‐enriched animals in this study, while housed without access to toys and other potentially stimulating materials, were housed in groups of three with access to social interactions. Normal animals reared in this manner have not been shown to have significant memory problems in a fear conditioning test (Lukkes et al., 2009). Although we did not observe modulatory effects of maternal care and type of postweaning environment on normal animals in the trace fear conditioning test, we cannot rule out the possibility that testing animals in a different cognitive task, such as Morris water maze or object recognition, tests previously shown to detect performance differences in low versus high maternal care offspring and enriched versus non‐enriched animals, would have produced different results (Bredy et al., 2003).

In contrast to what was observed in normal animals, the quality of maternal care and the complexity of the postweaning environment had significant effects on associative memory functioning in animals with early life Pb exposures. Consistent with previous reports from our lab (Anderson et al., 2016; Verma & Schneider, 2017), acquisition of the conditioned fear response was not impaired in either males or females in any of the Pb‐exposure groups in this study, but consolidation and longer‐term recall of the associative memory was negatively affected in PERI Pb‐exposed males and EPN Pb‐exposed females. Interestingly, this effect was only observed in animals with LMC mothers and that were housed in the non‐enriched postweaning environment. Additionally, for animals that lived in the non‐enriched environment postweaning, there was an advantage to having an HMC mother, such that these animals did not display the short‐term (at day 2 postconditioning) and long‐term (at day 10 postconditioning) associative memory impairments seen in similarly housed animals that had LMC mothers. These results suggest a potential protective effect of HMC in this non‐enriched housing condition. However, for animals living in the enriched environment, there were no differences in performance between animals with LMC and HMC mothers, suggesting a potential for environmental enrichment to reverse adverse influences from early life Pb exposure and rearing by an LMC mother.

The mechanisms underlying the sex‐specific effects of Pb exposures during different developmental windows on associative memory in the trace fear conditioning test are still not entirely clear and the subject of ongoing research. One possibility is that different timings of functional maturation of prefrontal cortex, dorsal hippocampus, and amygdala in males and females could potentially impact the influences of Pb exposure occurring during different developmental periods. Interactions between sex chromosomes and hormonal factors may also influence sex‐specific gene expression patterns, potentially in a brain region‐specific manner which could have a significant impact on brain regions like the hippocampus and frontal cortex that are known to be particularly sensitive to Pb exposure (see Singh et al., 2018 for review). Studies have also described various epigenetic and transcriptional effects from Pb exposure during gestation and the early postnatal period that differ depending on sex and development window of exposure, although the precise mechanisms underlying these effects remain uncertain (see Singh et al., 2018 for review). We previously showed that gene expression patterns differ in the hippocampus in males and females with Pb exposures at different developmental times (perinatal vs. early postnatal) (Schneider et al., 2012). Of particular interest were effects of these Pb exposures on the expression of numerous transcription factors and genes involved in plasticity.

Likewise, the precise mechanisms through which differences in the quality of maternal care and environmental enrichment may modify the effects of Pb on the brain are also not completely understood. Variations in the quality of maternal care are known to influence brain development, and in the rat, the amount of early postnatal maternal licking/grooming behavior influences epigenetic and transcriptional regulation, stress responsivity, and adult cognitive and behavioral outcomes (F. A. Champagne et al., 2003; D. L. Champagne et al., 2008). Rat pups that received low amounts of maternal licking and grooming early in life, in comparison to offspring of mothers who provided high amounts of licking and grooming, had decreased dendritic complexity as well as lower expression of synaptic markers and reduced spine density in the hippocampus CA1 and dentate gyrus in adulthood (Bagot et al., 2009; D. L. Champagne et al., 2008; Liu et al., 2000). Additionally, offspring of HMC mothers had increased hippocampal dendritic complexity, increased cholinergic innervation, and increased gene expression for *BDNF* and *NR2B, NR2A*, and *NR1* NMDA receptor subtypes in the hippocampus, compared with offspring of LMC mothers (D. L. Champagne et al., 2008; Liu et al., 2000). These morphological, neurochemical, and molecular changes were consistent with enhanced spatial learning and memory observed in offspring of HMC mothers or in offspring of LMC mothers cross‐fostered and reared by HMC mothers (Liu et al., 2000). Variations in maternal care have also been reported to produce tissue‐specific effects on gene expression in the hippocampus, amygdala, and medial prefrontal cortex (Meaney, 2001), the same brain regions involved in the trace fear conditioning task.

Synaptic plasticity, an important substrate for memory formation, is altered by reduced levels of maternal care (D. L. Champagne et al., 2008) as well as by early life Pb exposures. Developmental Pb exposure results in cognitive dysfunction as well as impairments in many of the same synaptic plasticity‐related mechanisms and expression of plasticity‐related genes (Schneider et al., 2012) that have been reported as disrupted in offspring of LMC mothers. Interestingly, postweaning environmental enrichment of offspring of LMC mothers reversed the effects of LMC on hippocampal synaptic density and NMDA and AMPA receptor expression as well reversed the effects of LMC on spatial learning and memory performance in the Morris water maze (Bredy et al., 2003, 2004).

Effects observed in this study could be the result of protection against detrimental Pb‐induced effects on CNS structure and function or stimulation of compensatory mechanisms that helped to reverse detrimental effects from the Pb exposures. We recently showed that living in an enriched postweaning environment reversed the trace fear conditioning memory deficits in EPN Pb‐exposed females and also reversed many of the Pb‐induced hippocampal CA1 transcriptional changes, as well as Pb exposure‐induced changes in upstream regulators, alternative splicing events, and long noncoding RNAs (Singh et al., 2022). Bredy et al. (2003) also showed that offspring of HMC mothers had enhanced spatial learning and object recognition memory compared to offspring of LMC mothers and that the differences in adult cognitive function related to maternal care differences were eliminated by postweaning housing of LMC offspring in an enriched environment. Environmental enrichment reversed changes in a number of genomic targets altered by reduced maternal care and we hypothesize that environmental enrichment and enhanced maternal care may also alter the Pb‐modified transcriptome. Studies of the effects of Pb exposure, maternal care, and environmental enrichment on the hippocampal, prefrontal cortex, and amygdala transcriptomes and genome‐wide methylation profiles are currently in progress in our lab and should provide more mechanistic insights.

In conclusion, this study shows that in the paradigm assessed, high‐quality maternal care may at least partially protect against some of the functional consequences of developmental Pb exposure and that living in an enriched environment may at least partially reverse adverse effects of developmental Pb exposure and low‐quality maternal care. There are, however, some limitations to this study. This work only assessed associative learning and memory and only utilized one task, trace fear conditioning. Additional studies need to be performed to assess the extent to which the current results are generalizable to other cognitive domains and behaviors adversely affected by developmental Pb exposure and as assessed by different tests. Additionally, this work was a behavioral study designed as the first to examine any potential interactive effects of maternal care characteristics, environmental enrichment on Pb‐induced cognitive dysfunction. Potential biological correlates of the current behavioral findings were not examined. As a result of these behavioral findings, additional studies are now needed to examine potential biological mechanisms that might underlie the observed effects and such studies are now in progress. Despite the above‐mentioned limitations, this line of research may have translational relevance. Children who grow up in low socioeconomic status environments are known to be at the highest risk for being exposed to Pb and suffering more severe adverse effects from this exposure. Risk factors of less enrichment in the home environment and less‐enriching parent–child interactions can often coexist in low SES communities (Bradley et al., 2001; Orr, 2003) and could contribute to enhanced neurotoxicity from Pb exposure. Child intervention programs, including supportive parenting interventions, have been shown to protect against poverty‐related adverse effects on children's brain structure and function (reviewed in Schneider, 2023). As we have recently suggested (Schneider, 2023), a variety of interventions including those aimed at enhancing the quality of the home environment and the amount and quality of parental interactions could be provided to Pb‐exposed children, and especially those Pb‐exposed children living in low SES households, to potentially stimulate plasticity and improve cognitive and educational outcomes in these children.

## AUTHOR CONTRIBUTIONS

**Jay S. Schneider**: Conceptualization; investigation; funding acquisition; writing—original draft; methodology; writing—review and editing; project administration. **Courtney Williams**: Investigation; writing—review and editing; methodology. **Shamaila Zafar**: Investigation. **Jaehyun Joo**: Formal analysis; writing—review and editing. **Blanca E. Himes**: Formal analysis; writing—review and editing.

## CONFLICT OF INTEREST STATEMENT

The authors declare no conflicts of interest.

### PEER REVIEW

The peer review history for this article is available at https://publons.com/publon/10.1002/brb3.70040.

## Supporting information




**Supplementary Table 1**. Summary statistics of learning (conditioning: percent freezing)
**Supplementary Table 2**. Summary statistics of associative memory function (percent freezing)
**Supplementary Figure 1**. Trends for percent freezing in the trace fear conditioning test on days 1, 2 and 10 post‐conditioning, indicative of associative memory functioning, in male and female offspring of high maternal care (HMC) or low maternal care (LMC) mothers and that lived in enriched or non‐enriched post‐weaning environments. Control = no Pb; EPN = early postnatal Pb exposure; PERI = perinatal Pb exposure.

## Data Availability

The data that support the findings of this study are available from the corresponding author upon reasonable request.
