# Supplementary material for: Influences of quality of maternal care and environmental enrichment on associative memory function in rats with early life lead exposure
Source: Brain Behav. 2024 Sep 18;14(9):e70040. doi: 10.1002/brb3.70040 (PMC11410876; doi:10.1002/brb3.70040)
Supplement: Supplementary file 1 — Supplementary Table 1. Summary statistics of learning (conditioning: percent freezing) Supplementary Table 2. Summary statistics of associative memory function (percent freezing) Supplementary Figure 1. Trends for percent freezing in the trace fear conditioning test on days 1, 2 and 10 post‐conditioning, indicative of associative memory functioning, in male and female offspring of high maternal care (HMC) or low maternal care (LMC) mothers and that lived in enriched or non‐enriched post‐weaning environments. Control = no Pb; EPN = early postnatal Pb exposure; PERI = perinatal Pb exposure. [file BRB3-14-e70040-s001.docx]

Influences of Quality of Maternal Care and Environmental Enrichment on Associative Memory Function in Rats with Early Life Lead Exposure.

J. S. Schneider^1,3^, C. Williams^1^, S. Zafar^1^, J. Joo^2,3^ and B. E. Himes^2,3^

^1^Department of Pathology and Genomic Medicine, Thomas Jefferson University, Philadelphia, PA 19107; ^2^Department of Biostatistics, Epidemiology and Informatics, Perelman School of Medicine, University of Pennsylvania, Philadelphia, PA 19104; ^3^Center of Excellence in Environmental Toxicology, Perelman School of Medicine, University of Pennsylvania, Philadelphia, PA 19104

**Supplementary Material**

**Supplementary Table 1.** Summary statistics of learning (conditioning: percent freezing).

|  |  |  | **Control** | | **EPN** | | **PERI** | | **EPN vs Control** | | | | **PERI vs Control** | | | | **PERI vs EPN** | | | |
| --- | --- | --- | --- | --- | --- | --- | --- | --- | --- | --- | --- | --- | --- | --- | --- | --- | --- | --- | --- | --- |
| **Sex** | **Enrichment Condition** | **Maternal Care** | **N** | **Mean (SE^†^)** | **N** | **Mean (SE)** | **N** | **Mean (SE)** | **Difference^‡^ (SE)** | ***t*** | ***df*** | ***P***^§^ | **Difference (SE)** | ***t*** | ***df*** | ***P*** | **Difference (SE)** | ***t*** | ***df*** | ***P*** |
| Male | Enriched | High | 8 | 82.61 (3.36) | 11 | 81.70 (2.86) | 7 | 84.06 (3.59) | -0.92 (4.41) | -0.21 | 195 | 0.98 | 1.44 (4.92) | 0.29 | 195 | 0.95 | 2.36 (4.59) | 0.51 | 195 | 0.86 |
|  |  | Low | 8 | 83.91 (3.36) | 11 | 78.94 (2.86) | 10 | 86.24 (3.00) | -4.97 (4.41) | -1.13 | 195 | 0.50 | 2.34 (4.51) | 0.52 | 195 | 0.86 | 7.30 (4.15) | 1.76 | 195 | 0.19 |
|  | Non-enriched | High | 6 | 81.32 (3.88) | 12 | 78.50 (2.74) | 10 | 78.97 (3.00) | -2.82 (4.75) | -0.59 | 195 | 0.82 | -2.34 (4.90) | -0.48 | 195 | 0.88 | 0.48 (4.07) | 0.12 | 195 | 0.99 |
|  |  | Low | 10 | 80.77 (3.00) | 10 | 82.91 (3.00) | 9 | 74.61 (3.17) | 2.14 (4.25) | 0.5 | 195 | 0.87 | -6.16 (4.36) | -1.41 | 195 | 0.34 | -8.30 (4.36) | -1.90 | 195 | 0.14 |
| Female | Enriched | High | 7 | 86.58 (3.59) | 13 | 82.48 (2.63) | 8 | 77.34 (3.36) | -4.10 (4.45) | -0.92 | 195 | 0.63 | -9.24 (4.92) | -1.88 | 195 | 0.15 | -5.14 (4.27) | -1.20 | 195 | 0.45 |
|  |  | Low | 8 | 79.85 (3.36) | 9 | 78.86 (3.17) | 8 | 81.20 (3.36) | -0.99 (4.62) | -0.22 | 195 | 0.98 | 1.34 (4.75) | 0.28 | 195 | 0.96 | 2.34 (4.62) | 0.51 | 195 | 0.87 |
|  | Non-enriched | High | 6 | 80.64 (3.88) | 11 | 76.53 (2.86) | 10 | 76.42 (3.00) | -4.11 (4.82) | -0.85 | 195 | 0.67 | -4.22 (4.90) | -0.86 | 195 | 0.66 | -0.11 (4.15) | -0.03 | 195 | 1.00 |
|  |  | Low | 8 | 81.58 (3.36) | 9 | 81.48 (3.17) | 10 | 77.93 (3.00) | -0.10 (4.62) | -0.02 | 195 | 1.00 | -3.65 (4.51) | -0.81 | 195 | 0.70 | -3.55 (4.36) | -0.81 | 195 | 0.70 |

^†^Standard Error; ^‡^A vs B = A – B; ^§^Adjusted P-value; t = t statistic; df = degrees of freedom. Numbers represent freezing response (i.e., mean (SE) percent of time freezing)

**Supplementary Table 2.** Summary statistics of associative memory function (percent freezing).

|  |  |  | **Control** | | **EPN** | | **PERI** | | **EPN vs Control** | | | | **PERI vs Control** | | | | **PERI vs EPN** | | | |
| --- | --- | --- | --- | --- | --- | --- | --- | --- | --- | --- | --- | --- | --- | --- | --- | --- | --- | --- | --- | --- |
| Sex | **Environmental Enrichment** | **Maternal Care** | **N** | **Mean (SE^†^)** | **N** | **Mean (SE)** | **N** | **Mean (SE)** | **Difference^‡^ (SE)** | ***t*** | ***df*** | ***P***^§^ | **Difference (SE)** | ***t*** | ***df*** | ***P*** | **Difference (SE)** | ***t*** | ***df*** | ***P*** |
| Male | Enriched | High | 8 | 97.82 (3.84) | 11 | 96.61 (3.29) | 7 | 99.00 (4.10) | -1.21 (5.05) | -0.24 | 195 | 0.97 | 1.18 (5.62) | 0.21 | 195 | 0.98 | 2.39 (5.26) | 0.46 | 195 | 0.89 |
|  |  | Low | 8 | 96.00 (3.84) | 11 | 96.41 (3.30) | 10 | 89.40 (3.43) | 0.41 (5.06) | 0.08 | 195 | 1.00 | -6.60 (5.15) | -1.28 | 195 | 0.41 | -7.01 (4.76) | -1.47 | 195 | 0.31 |
|  | Non-enriched | High | 6 | 97.73 (4.72) | 12 | 89.12 (3.14) | 10 | 92.83 (3.45) | -8.61 (5.67) | -1.52 | 195 | 0.28 | -4.91 (5.76) | -0.85 | 195 | 0.67 | 3.70 (4.67) | 0.79 | 195 | 0.71 |
|  |  | Low | 10 | 94.97 (3.43) | 10 | 93.83 (3.43) | 9 | 71.46 (3.62) | -1.14 (4.85) | -0.24 | 195 | 0.97 | -23.50 (4.98) | -4.72 | 195 | 1.2e-05 | -22.36 (4.99) | -4.48 | 195 | 3.7e-05 |
| Female | Enriched | High | 7 | 97.12 (4.10) | 13 | 92.40 (3.02) | 8 | 97.98 (3.84) | -4.72 (5.09) | -0.93 | 195 | 0.62 | 0.86 (5.62) | 0.15 | 195 | 0.99 | 5.58 (4.88) | 1.14 | 195 | 0.49 |
|  |  | Low | 8 | 93.89 (3.85) | 9 | 94.04 (3.62) | 8 | 97.21 (3.83) | 0.14 (5.28) | 0.03 | 195 | 1.00 | 3.31 (5.43) | 0.61 | 195 | 0.82 | 3.17 (5.27) | 0.60 | 195 | 0.82 |
|  | Non-enriched | High | 6 | 98.26 (4.44) | 11 | 88.16 (3.27) | 10 | 87.45 (3.64) | -10.10 (5.52) | -1.83 | 195 | 0.16 | -10.80 (5.76) | -1.88 | 195 | 0.15 | -0.71 (4.90) | -0.14 | 195 | 0.99 |
|  |  | Low | 8 | 96.81 (3.84) | 9 | 76.09 (3.62) | 10 | 87.75 (3.48) | -20.72 (5.27) | -3.93 | 195 | 3.5e-04 | -7.06 (5.18) | -1.36 | 195 | 0.36 | 13.66 (5.02) | 2.72 | 195 | 0.02 |

^†^Standard Error; ^‡^A vs B = A – B; ^§^Adjusted P-value; t = t statistic; df = degrees of freedom. Numbers represent freezing response (i.e., mean (SE) percent of time freezing).


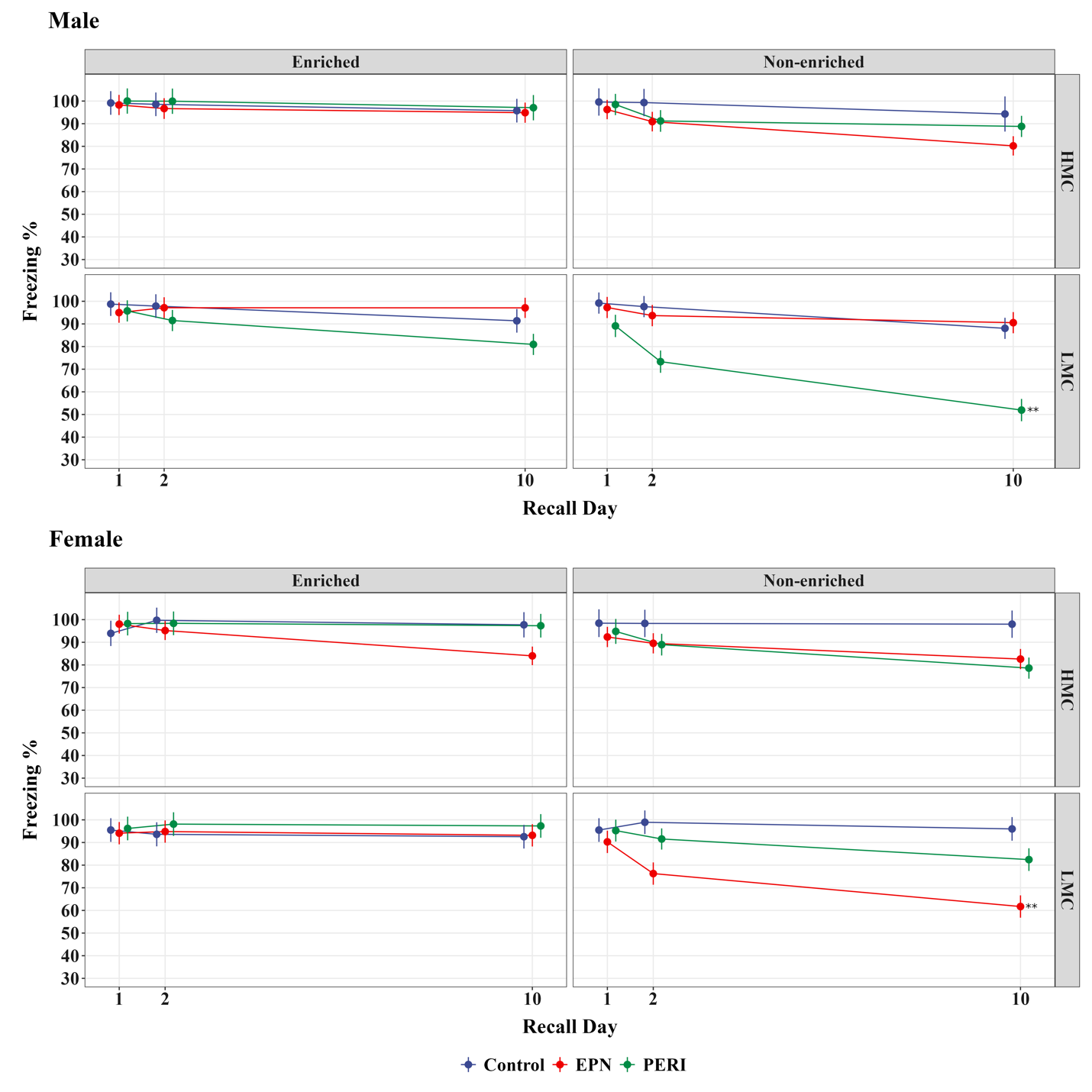


**Supplementary Figure 1.** Trends for percent freezing in the trace fear conditioning test on days 1, 2 and 10 post-conditioning, indicative of associative memory functioning, in male and female offspring of high maternal care (HMC) or low maternal care (LMC) mothers and that lived in enriched or non-enriched post-weaning environments. Control = no Pb; EPN = early postnatal Pb exposure; PERI = perinatal Pb exposure.
